# ﻿Six new species of micropterous *Paederus* (Coleoptera, Staphylinidae, Paederinae) from China

**DOI:** 10.3897/zookeys.1221.135891

**Published:** 2024-12-13

**Authors:** Yi Yang, Sophie Chen, Zhong Peng

**Affiliations:** 1 College of Life Sciences, Shanghai Normal University, 100 Guilin Road, 1st Educational Building 323 Room, Shanghai, 200234, China Shanghai Normal University Shanghai China; 2 Shanghai American School, 1600 Lingbai Road, Shanghai, 200120, China Shanghai American School Shanghai China

**Keywords:** New species, rove beetles, taxonomic key

## Abstract

Six new species of the genus *Paederus* Fabricius, 1775 from China are described: *P.chentangus***sp. nov.** (Xizang: Chentang), *P.mirus***sp. nov.** (Xizang: Xiayadong), *P.songi***sp. nov.** (Chongqing: Polaoxiang), *P.trispinosus***sp. nov.** (Hubei: Houhe), P. (Harpopaederus) yei**sp. nov.** (Hubei: Cuijia’ao), and *P.zhaoi***sp. nov.** (Zhejiang: Majian). A key to the micropterous *Paederus* species of mainland China is given.

## ﻿Introduction

The genus *Paederus* Fabricius, 1775 was previously represented in mainland China by 55 known species and in Taiwan by 25 known species (Assing 2017, 2020, 2022). According to a key provided by Li et al. (2016), 31 micropterous species of *Paederus* were previously reported from mainland China. In recent years, three additional species have been described (Assing 2017; Cheng and Peng 2019; Assing 2020), thus raising the total number of micropterous species known from mainland China to 34. The *Paederus* fauna of over 50 mountain ranges in China has not been examined and most of these ranges have suitable habitats for the micropterous *Paederus*, suggesting that the true diversity of the genus is far greater than currently known.

A study of the micropterous *Paederus* material of mainland China yielded six new species.

## ﻿Materials and methods

The genitalia and other dissected parts were mounted on plastic slides and attached to the same pin as the respective specimens. Photographs were taken with a Canon EOS 7D camera with a MP-E 65 mm macro lens or with a Canon G9 camera mounted on an Olympus CX 31 microscope.

The following abbreviations are used in the text, with all measurements in millimeters:

Body length (**BL**) from the anterior margin of the labrum to the abdominal apex; forebody length (**FL**) from the anterior margin of the labrum to the posterior margin of the elytra; head length (**HL**) from the anterior clypeal margin to the occipital constriction; maximum width of head (**HW**); length of antenna (**AnL**); length of pronotum (**PL**) along midline; maximum width of pronotum (**PW**); elytral length (**EL**) at the suture from the apex of the scutellum to the posterior margin of the elytra (at the sutural angles); maximum width of the elytra (**EW**); maximum width of abdomen (**AW**); length of aedeagus (**AL**) from the apex of the dorsal plate or the parameres (whichever forms the apex of the aedeagus) to the base of the aedeagal capsule.

All material treated in this paper is deposited in the Insect Collection of Shanghai Normal University, Shanghai, China (**SNUC**). The type labels are cited in the original spelling; different labels are separated by slashes.

## ﻿Results

### Paederus chentangus

Taxon classificationAnimaliaColeopteraStaphylinidae

Yang & Peng sp. nov.

75596528-A05A-5156-80AF-CA4152252D38

https://zoobank.org/1436E3DD-0963-4800-BE95-CAD08EC18C76

Figs 1A , 2 , 8 , 9

#### Type material.

***Holotype.*** China – **Xizang Prov.** • ♂; glued on a card with two labels as follows: “China: Xizang Prov., Dingjie County, Chentang Town, Xiuxiongma Vill., alt. 27°54'11"N, 87°22'42"E, 2700–3000 m, 25.VI.2021, Peng, Yin & Zhang leg.” “Holotype: *Paederuschentangus* sp. n., Yang & Peng des. 2024” [red handwritten label]; SNUC. ***Paratypes.*** China – **Xizang Prov.** • 8 ♂♂, 12 ♀♀; Dingjie County, Chentang Town, Xiuxiongma Vill., alt. 27°54'11"N, 87°22'42"E, 2700–3000 m, 25.VI.2021, Peng, Yin & Zhang leg; SNUC • 4 ♂♂, 12 ♀♀; Dingjie County, Chentang Town, Ganma Zangbu, 27°51'38"N, 87°24'59"E, alt. 2300 m, 30.VII.2022, Peng, Song, Yin & Zhang leg; SNUC • 3 ♀♀; Dingjie County, Chentang Town, Ganma Zangbu, 27°51'50"N, 87°24'24"E, alt. 2400 m, 28.VI.2021, Z. Peng leg; SNUC.

#### Description.

Measurements (in mm) and ratios: BL: 9.39–11.22; FL: 4.43–5.00; HL: 1.25–1.37; HW: 1.47–1.57; AnL: 2.78–3.17; PL: 1.34–1.57; PW: 1.42–1.50; EL: 1.05–1.12; EW: 1.49–1.62; AW: 1.62–1.69; AL: 1.72–1.74; HL/HW: 0.84–0.87; HW/PW: 0.98–1.05; HL/PL: 0.87–0.93; PL/PW: 0.95–1.05; EL/PL: 0.71–0.78; diameter of eye: 0.37–0.42.

**Figure 1.**
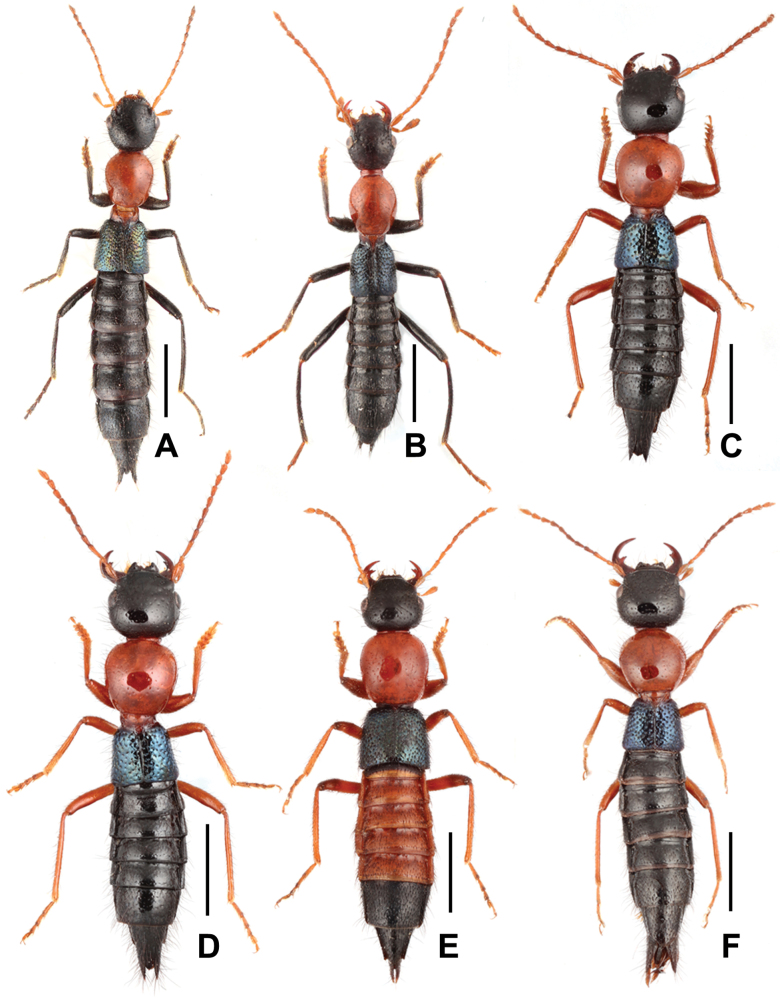
Habitus of *Paederus* species **A***P.chentangus***B***P.mirus***C***P.songi***D***P.trispinosus***E***P.yei***F***P.zhaoi*. Scale bars: 2.0 mm.

Habitus as in Figs 1A, 8. Coloration: head and abdomen black; antennae light brown, sometimes antennomeres 4–11 infuscate; pronotum red; elytra black with bluish-green hue; legs black with blackish-brown tarsi.

**Figure 2.**
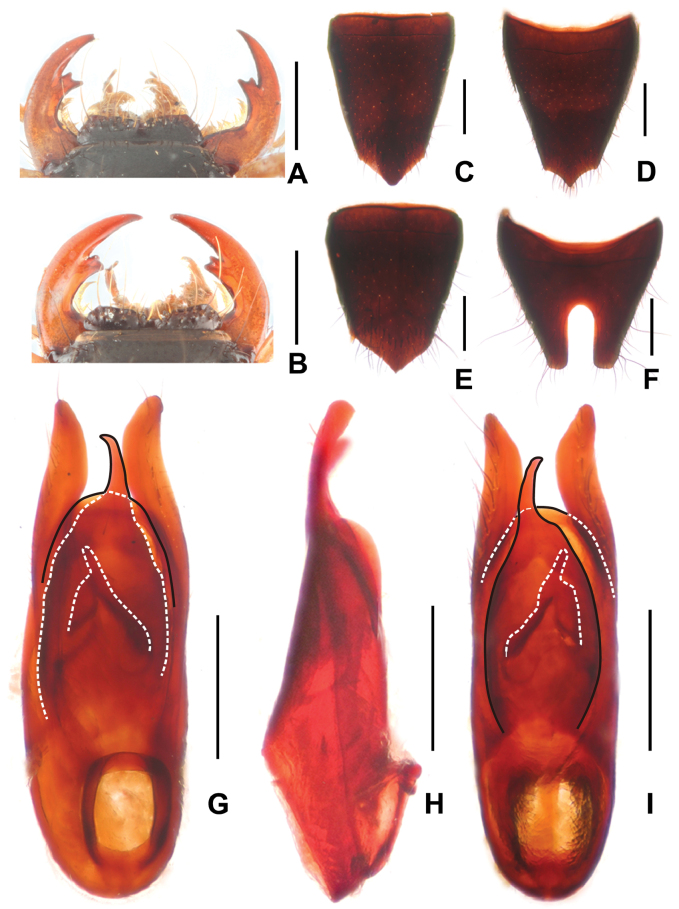
*Paederuschentangus***A** female mouthparts **B** male mouthparts **C** female tergite VIII **D** female sternite VIII **E** male tergite VIII **F** male sternite VIII **G** aedeagus in ventral view **H** aedeagus in lateral view **I** aedeagus in dorsal view. Scale bars: 0.5 mm.

Head (Fig. 1A) transverse, widest across eyes; punctation coarse and moderately dense; interstices glossy. Eyes distinctly convex. Antennae slender, antennomere 4 approximately 3.8 times as long as broad and antennomere 10 nearly twice as long as broad. Mandibles (Fig. 2A, B) each with apically bifid molar tooth, without evident sexual dimorphism.

Pronotum (Fig. 1A) weakly transverse or as long as broad, strongly convex in cross section; punctures distinctly sparser and shallower than that of head.

Elytra (Fig. 1A) nearly trapeziform; punctation coarse, well defined, and dense. Hind wings reduced. Metatarsomere I shorter than combined length of metatarsomeres II and III.

Abdomen slightly broader than elytra; punctation dense; interstices with distinctly transverse microsculpture; posterior margin of tergite VII without palisade fringe.

**Male.** Anterior margin of labrum (Fig. 2B) in middle with semicircular median excision; posterior margin of tergite VIII (Fig. 2E) pointed in middle; sternite VII unmodified; sternite VIII (Fig. 2F) with deep posterior excision, this excision approximately 0.4 times as long as sternite VIII; aedeagus as in Fig. 2G–I, ventral plate apically convex in ventral view; dorsal plate asymmetric, apically acute and not reaching apices of parameres in ventral view; parameres weakly asymmetric and apically distinctly curved in lateral view; internal sac with single distinctive sclerotized spine.

**Female.** Anterior margin of labrum (Fig. 2A) in middle with shallow median excision. Posterior margin of tergite VIII (Fig. 2C) strongly convex; posterior margin of sternite VIII (Fig. 2D) obtusely pointed in middle.

#### Distribution and biological notes.

The species was found in three localities in the Chentang area, to the south of Dingjie, southern Xizang. The specimens were sifted from moss, grass roots and loose gravel in shrub habitats at altitudes of 2300–3000 m (Figs 8, 9).

#### Etymology.

The species is named after its type locality (Chentang).

#### Comparative notes.

Based on the sexual characters and the external characters, closer affiliations with other *Paederus* species from Xizang are not evident. However, the highly similar male sexual characters, particularly the similarly derived morphology of the aedeagus, suggest that *P.chentangus* is very closely related to *P.megascutum* Willers, 1999 from Nepal. It differs from *P.megascutum* by the shape of the head (weakly transverse in *P.megascutum*), by the shorter elytra, and particularly by the stouter parameres and the curved ventral plate of the aedeagus in ventral view. For illustrations of *P.megascutum*, see Willers (1999: figs 7, I4, II4).

### Paederus mirus

Taxon classificationAnimaliaColeopteraStaphylinidae

Yang & Peng sp. nov.

FB473D15-CAA6-5922-B226-0D1C8C0152E2

https://zoobank.org/D8356E73-3E02-40F7-8609-F0D60478A008

Figs 1B , 3 , 10

#### Type material.

***Holotype.*** China – **Xizang Prov.** • ♂; glued on a card with two labels as follows: “China: Xizang Prov., Yadong County, Xiayadong, 27°23'48"N, 88°50'02"E, alt. 3000 m, 01.VIII.2021, Peng, Yin & Zhang leg.” “HOLOTYPE: *Paederusmirus* sp. n., Yang & Peng des. 2024” [red handwritten label]; SNUC. ***Paratypes.*** China – **Xizang Prov.** • 3 ♀♀: Yadong County, Xiayadong, 27°23'48"N, 88°50'02"E, alt. 3000 m, 01.VIII.2021, Peng, Yin & Zhang leg; SNUC • 1 ♀: Yadong County, Xiayadong, 27°23'48"N, 88°50'02"E, alt. 2750 m, 10.VIII.2010, Wen-Xuan Bi leg; SNUC.

#### Description.

Measurements (in mm) and ratios: BL 9.10–9.45, FL 4.42–4.77, HL 1.26–1.32, HW 1.29–1.35, AnL: 3.24–3.78; PL: 1.50–1.57; PW: 1.26–1.29; EL: 1.01–1.09; EW: 1.24–1.27; AW: 1.51–1.59; AL: 0.95; HL/HW: 0.96–0.98; HW/PW: 1.02–1.05; HL/PL: 0.84–0.85; PL/PW: 1.19–1.21; EL/PL: 0.67–0.69; diameter of eye: 0.32–0.33.

Habitus as in Fig. 1B. Coloration: head and abdomen black; antennae light brown; pronotum red; elytra black with faint bluish hue; legs black with dark brown tarsi.

**Figure 3.**
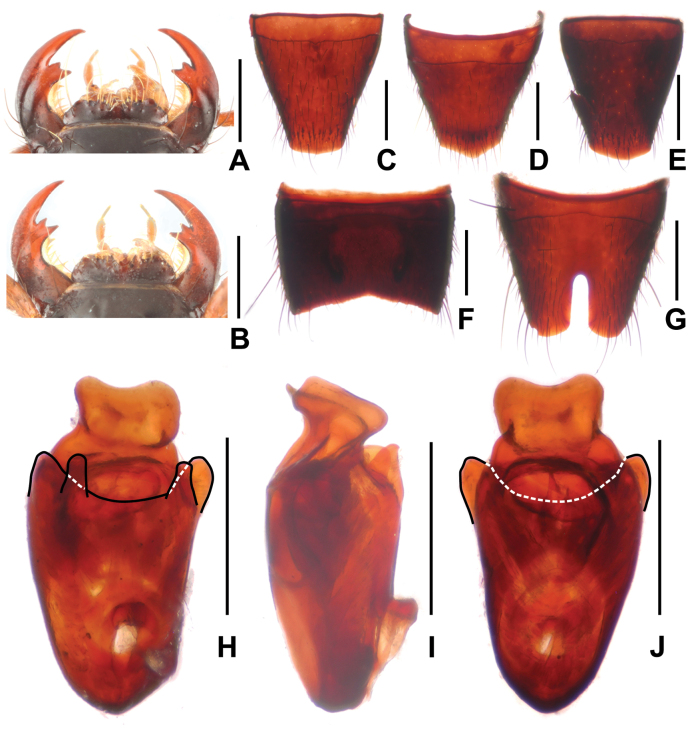
*Paederusmirus***A** female mouthparts **B** male mouthparts **C** female tergite VIII **D** female sternite VIII **E** male tergite VIII **F** male sternite VII **G** male sternite VIII **H** aedeagus in ventral view **I** aedeagus in lateral view **J** aedeagus in dorsal view. Scale bars: 0.5 mm.

Head (Fig. 1B) weakly transverse, widest across eyes; punctation distinctly coarse and sparse; interstices glossy. Eyes distinctly convex. Antennae distinctly slender, antennomere 4 nearly four times as long as broad and antennomere 10 nearly twice as long as broad. Labrum (Fig. 3A, B) without evident sexual dimorphism, anterior margin in middle with U-shaped median excision, with small projection on either side of median excision. Mandibles (Fig. 3A, B) each with apically bifid molar tooth, without sexual dimorphism.

Pronotum (Fig. 1B) nearly oviform, strongly convex in cross section; punctures sparser and shallower than on head.

Elytra (Fig. 1B) slightly slender, humeral angles obsolete; punctation coarse, well defined, and dense. Hind wings reduced. Metatarsomere I as long as combined length of metatarsomeres II and III.

Abdomen broader than elytra; punctation dense; interstices with shallow microsculpture; posterior margin of tergite VII without palisade fringe; posterior margin of tergite VIII (Fig. 3C, E) weakly convex, shape subject to some variation, but without evident sexual dimorphism.

**Male.** Sternites III–VI unmodified; sternite VII (Fig. 3F) strongly transverse, with deep median impression without modified pubescence, posterior margin broadly concave; sternite VIII (Fig. 3G) with deep posterior excision, this excision approximately 0.4 times as long as sternite VIII; aedeagus as in Fig. 3H–J and strongly derived; ventral plate nearly truncate; dorsal plate weakly asymmetric, apically bifid and conspicuous; parameres very short; internal sac without sclerotized spine.

**Female.** Posterior margin of sternite VIII (Fig. 3D) convex.

#### Distribution and biological notes.

The species was discovered in two localities situated to southern Yadong, southern Xizang. Some specimens were sifted from moss and leaf litter in montane primary mixed and coniferous forests at an altitude of 3000 m (Fig. 10).

#### Etymology.

The specific epithet *mirus* means “strange, wonderful”, referring to the shape of the aedeagus.

#### Comparative notes.

*Paederusmirus* is characterized particularly by the distinctive shape of the aedeagus and additionally by the shape and chaetotaxy of the male sternite VII, as well as by the slender habitus and antennae. Based on the sexual characters, closer affiliations with other *Paederus* species are not evident.

### Paederus songi

Taxon classificationAnimaliaColeopteraStaphylinidae

Yang & Peng sp. nov.

2B2DB6C0-895E-574E-BF51-05EA6229C457

https://zoobank.org/ED02AC7E-1878-4FD6-B4E3-D67865AB93DA

Figs 1C , 4

#### Type material.

***Holotype.*** China – **Chongqing** • ♂; glued on a card with two labels as follows: “China: Chongqing City, Shizhu County, Huangshui Town, Polaoxiang, Near Dafengbao, 30°13'04"N, 108°24'50"E, alt. 1550 m, 27.IX.2023, Xiao-Bin Song leg.” “HOLOTYPE: *Paederussongi* sp. n., Yang & Peng des. 2024” [red handwritten label]; SNUC. ***Paratypes.*** China – **Chongqing** • 1♀; Shizhu County, Huangshui Town, Polaoxiang, Near Dafengbao, 30°13'04"N, 108°24'50"E, alt. 1550 m, 27.IX.2023, Xiao-Bin Song leg; SNUC.

#### Description.

Measurements (in mm) and ratios: BL 8.11–9.55, FL 4.45–5.02, HL 1.49–1.52, HW 1.54–1.71, AnL: 2.94–3.31; PL: 1.54–1.72; PW: 1.59–1.68; EL: 0.95–1.02; EW: 1.55–1.60; AW: 1.73–1.79; AL: 2.25; HL/HW: 0.89–0.97; HW/PW: 0.97–1.02; HL/PL: 0.88–0.97; PL/PW: 0.97–1.02; EL/PL: 0.59–0.62; diameter of eye: 0.38–0.45.

Habitus as in Fig. 1C. Coloration: head and abdomen black; antennae brown to light brown; pronotum red; elytra black with distinctly bluish hue; legs brown with paler tarsi.

Head (Fig. 1C) transverse, widest across eyes; punctation coarse and sparse; interstices glossy. Eyes distinctly convex. Antennae slender, antennomere 4 approximately 3.4 times as long as broad and antennomere 10 nearly twice as long as broad.

Pronotum (Fig. 1C) nearly as long as broad, strongly convex in cross section; punctures slightly sparser than on head.

Elytra (Fig. 1C) trapeziform; punctation distinctly coarse, well defined, and moderately dense. Hind wings reduced. Metatarsomere I slightly shorter than combined length of metatarsomeres II and III.

Abdomen broader than elytra; punctation dense; interstices with distinctly transverse microsculpture; posterior margin of tergite VII without palisade fringe.

**Figure 4.**
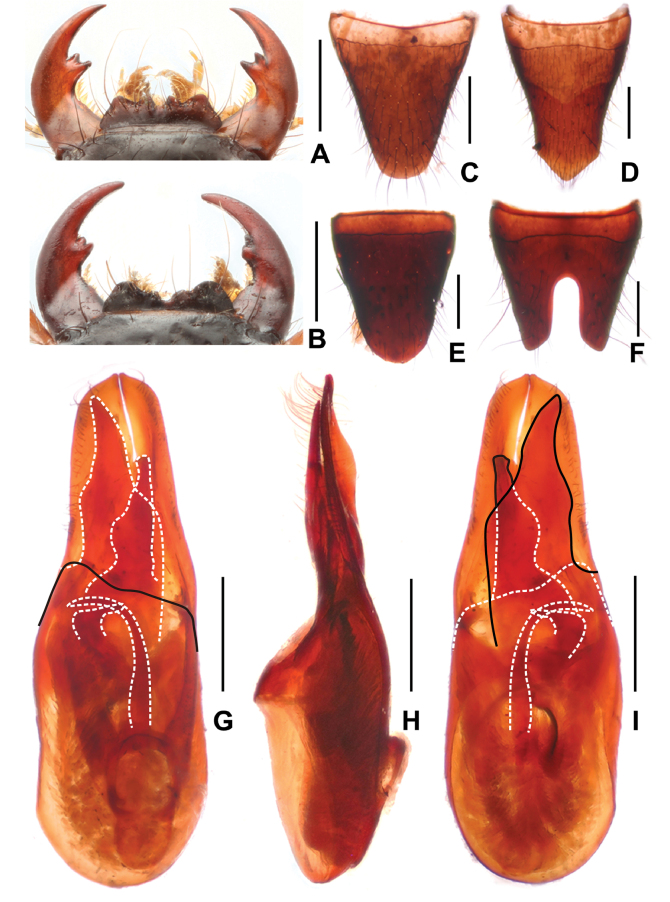
*Paederussongi***A** female mouthparts **B** male mouthparts **C** female tergite VIII **D** female sternite VIII **E** male tergite VIII **F** male sternite VIII **G** aedeagus in ventral view **H** aedeagus in lateral view **I** aedeagus in dorsal view. Scale bars: 0.5 mm.

**Male.** Mandibles (Fig. 4B) each with weakly bifid molar tooth at apex. Labrum (Fig. 4B) with distinctly concave anterior margin, with U-shaped median excision and with broad lateral projection on either side. Posterior margin of tergite VIII (Fig. 4E) convex; sternite VII unmodified; sternite VIII (Fig. 4F) with deep posterior excision, this excision approximately 0.4 times as long as sternite VIII; aedeagus as in Fig. 4G–I, ventral plate asymmetric in ventral view; dorsal plate asymmetric, apically acute in dorsal view and not reaching apices of parameres; parameres asymmetric and weakly curved in lateral view; internal sac with pair of long sclerotized spines and single very short sclerotized spine.

**Female.** Mandibles (Fig. 4A) each with bifid molar tooth of similar shape. Labrum (Fig. 4A) with U-shaped median excision and with broad lateral projection on either side, as well as a small projection on either side of median excision. Tergite VIII (Fig. 4C) oblong, with convex posterior margin; posterior margin of sternite VIII (Fig. 4D) strongly convex.

#### Distribution and biological notes.

The type locality is in northeastern Shizhu, eastern Chongqing. The specimens were sifted from moist leaf litter and roots in a secondary deciduous forest with bamboo at an altitude of 1550 m (Song pers. comm.). The paratype is teneral.

#### Etymology.

The species is named after Xiao-Bin Song, who collected the type specimens. He is a renowned specialist on mainly Palaearctic Paussinae.

#### Comparative notes.

The external and particularly the male sexual characters leave no doubt that this species belongs to the *P.biacutus* group. Among the species of this group, it appears to be most closely allied to *P.sinisterobliquus* Li, Zhou & Solodovnikov, 2013, with which it shares the similar morphology of the aedeagus. It is distinguished from *P.sinisterobliquus* by slightly larger body size, by the stouter pronotum, by three distinctly sclerotized spines of the internal sac and the larger parameres of the aedeagus, as well as by the shape of the female sternite VIII.

### Paederus trispinosus

Taxon classificationAnimaliaColeopteraStaphylinidae

Yang & Peng sp. nov.

9620B9E8-39A2-50F5-BF5C-B27BCD4D199C

https://zoobank.org/5AB15EEF-ED8D-461D-9696-F78D262EDFCE

Figs 1D , 5 , 11

#### Type material.

***Holotype.*** China – **Hubei Prov.** • ♂; glued on a card with two labels as follows: “China: Hubei Prov., Wufeng County, Houhe N.R., 30°11'53"N, 110°35'40"E, alt. 1480 m, 12.V.2020, Wen-Xuan Bi leg.” “HOLOTYPE: *Paederustrispinosus* sp. n., Yang & Peng des. 2024” [red handwritten label]; SNUC. ***Paratypes.*** China – **Hubei Prov.** •4 ♂♂, 2 ♀♀; Wufeng County, Houhe N.R., 30°11'53"N, 110°35'40"E, alt. 1480 m, 12.V.2020, Wen-Xuan Bi leg; SNUC • 1 ♂; Wufeng County, Houhe N.R., 30°05'10"N, 110°33'04"E, alt. 1150 m, 30.IV.2004, Li-Zhen Li leg; SNUC.

#### Description.

Measurements (in mm) and ratios: BL 9.11–9.67, FL 4.56–4.91, HL: 1.35–1.54; HW: 1.49–1.69; AnL: 3.08–3.22; PL: 1.49–1.67; PW: 1.50–1.67; EL: 1.00–1.10; EW: 1.47–1.54; AW: 1.67–1.74; AL: 2.14–2.17; HL/HW: 0.90–0.93; HW/PW: 0.99–1.02; HL/PL: 0.90–0.93; PL/PW: 0.97–1.01; EL/PL: 0.65–0.68; diameter of eye: 0.38–0.43.

Habitus as in Fig. 1D. Coloration: head and abdomen black; antennae brown, sometimes antennomeres 4−8 infuscate; pronotum red; elytra black with distinctly bluish hue; legs with brown femora, and with brown to light brown tibiae and tarsi.

**Figure 5.**
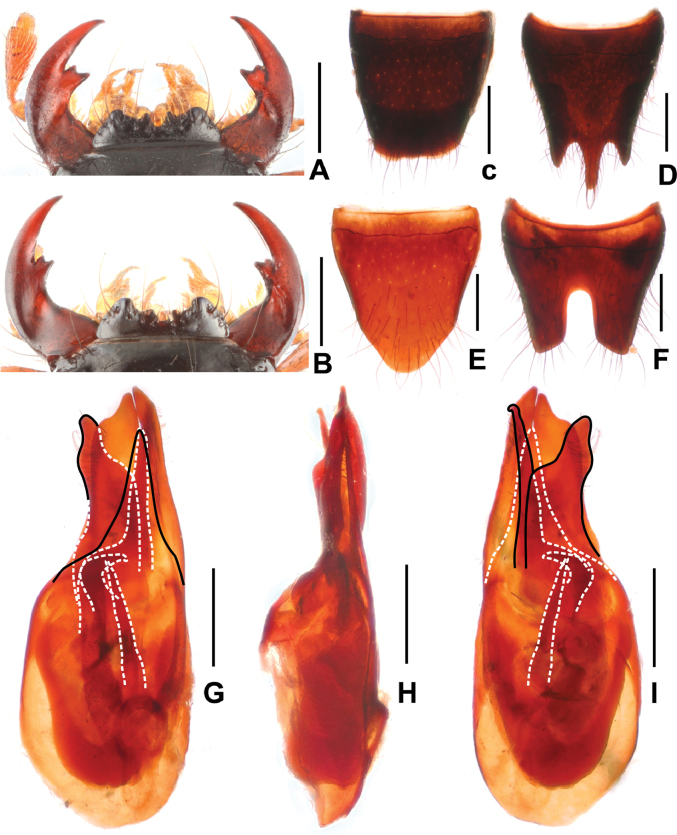
*Paederustrispinosus***A** female mouthparts **B** male mouthparts **C** female tergite VIII **D** female sternite VIII **E** male tergite VIII **F** male sternite VIII **G** aedeagus in ventral view **H** aedeagus in lateral view **I** aedeagus in dorsal view. Scale bars: 0.5 mm.

Head (Fig. 1D) transverse, widest across eyes; punctation coarse and sparse; interstices glossy. Eyes distinctly convex. Antennae slender, antennomere 4 approximately 3.3 times as long as broad and antennomere 10 1.8 times as long as broad. Mandibles (Fig. 5A, B) each with apically bifid molar tooth, without evident sexual dimorphism.

Pronotum (Fig. 1D) nearly as long as broad, strongly convex in cross section; punctures slightly sparser than on head.

Elytra (Fig. 1D) trapeziform; punctation distinctly coarse, defined and moderately dense. Hind wings reduced. Metatarsomere I as long as combined length of metatarsomeres II and III.

Abdomen distinctly broader than elytra; punctation dense; interstices with distinctly transverse microsculpture; posterior margin of tergite VII without palisade fringe.

**Male.** Labrum (Fig. 5B) with distinctly concave anterior margin, with U-shaped median excision and with large lateral projection on either side, as well as indistinct projection on either side of median excision. Posterior margin of tergite VIII (Fig. 5E) strongly convex; sternite VII unmodified; sternite VIII (Fig. 5F) with deep posterior excision, this excision approximately 0.3 times as long as sternite VIII; aedeagus as in Fig. 5G–I, ventral plate long and apically acute in ventral view; dorsal plate asymmetric and strongly sclerotized, with obtusely acute apical portion and not reaching apices of parameres; parameres distinctly asymmetric and apically straight in lateral view; internal sac with three distinctive sclerotized spines.

**Female.** Labrum (Fig. 5A) with U-shaped median excision and with broad lateral projection on either side, as well as a small projection on either side of median excision. Posterior margin of tergite VIII (Fig. 5C) weakly convex; posterior margin of sternite VIII distinctly trifurcate as in Fig. 5D.

#### Distribution and biological notes.

The species was found in two localities in the Houhe Natural Reserve, to western Wufeng, Hubei. Some specimens were sifted from leaf litter, grass roots and the soil along a forest path at an altitude of 1480 m (Fig. 11).

#### Etymology.

The speciﬁc epithet of this new species consists of the Latin sufﬁx *tri*- (which means “three”) and the Latin adjective *spinosus* (which means “spiny”). The name (a Latin adjective) refers to three distinctive sclerotized spines in the internal sac of the aedeagus.

#### Comparative notes.

The external and particularly the male sexual characters leave no doubt that this species belongs to the *P.biacutus* group. This new species is distinguished from other species of this group by the shape of female tergite VIII and the morphology of the aedeagus (the distinctly asymmetric dorsal plate and parameres, as well as two long sclerotized spines and one hooked sclerotized spine in the internal sac).

### Paederus (Harpopaederus) yei

Taxon classificationAnimaliaColeopteraStaphylinidae

Yang & Peng sp. nov.

7DC62C61-90CB-52F7-A0B0-66929035704A

https://zoobank.org/54D298FE-9877-443A-8C9A-CD6DF6CB782B

Figs 1E , 6

#### Type material.

***Holotype.*** China – **Hubei Prov.** • ♂; glued on a card with two labels as follows: “China: Hubei Prov., Baokang County, Cuijia’ao, 31.72°N, 111.13°E, alt. 1550 m, 31.VII.2021, Mao Ye leg.” “HOLOTYPE: *Paederus (Harpopaederus) Paederusyei* sp. n., Yang & Peng des. 2024” [red handwritten label]; SNUC. ***Paratypes.*** China – **Hubei Prov.** • 6 ♂♂, 5 ♀♀; Baokang County, Cuijia’ao, 31.72°N, 111.13°E, alt. 1550 m, 31.VII.2021, Mao Ye leg; SNUC • 3 ♂♂: Baokang County, Longping, alt. 1100 m, 15.VII.2017, Lu Qiu leg; SNUC.

#### Description.

Measurements (in mm) and ratios: BL 9.43–10.02, FL 4.46–5.01, HL 1.20–1.25, HW 1.44–1.54, AnL: 2.67–2.79; PL: 1.49–1.67; PW: 1.49–1.69; EL: 1.14–1.20; EW: 1.64–1.74; AW: 1.62–1.74; AL: 1.94–1.99; HL/HW: 0.81–0.83; HW/PW: 0.91–0.97; HL/PL: 0.75–0.81; PL/PW: 0.98–1.01; EL/PL: 0.72–0.76; diameter of eye: 0.30–0.38.

Habitus as in Fig. 1E. Coloration: head and apex of abdomen black; labrum blackish brown; antennae brown, with the four basal and two apical segments yellowish brown; pronotum red; elytra black with faint bluish hue; first four abdominal segments reddish brown; legs with dark brown femora and protibiae, and with brown to light brown meso- and metatibiae and tarsi.

**Figure 6.**
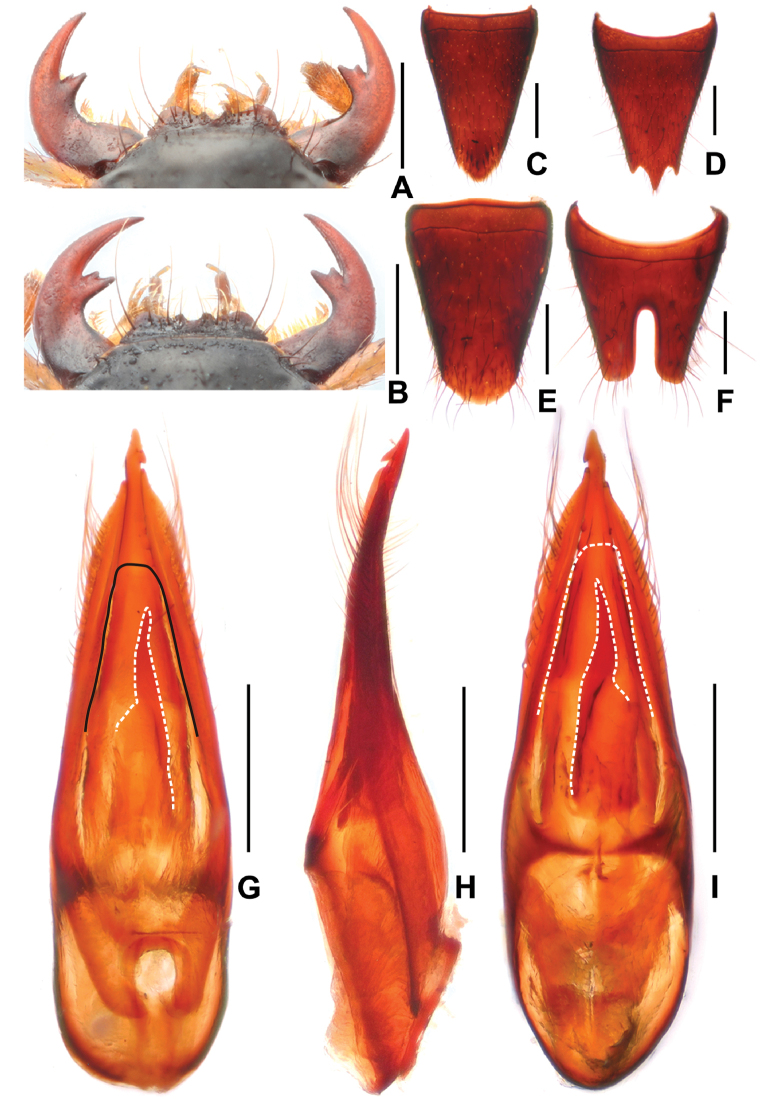
*Paederusyei***A** female mouthparts **B** male mouthparts **C** female tergite VIII **D** female sternite VIII **E** male tergite VIII **F** male sternite VIII **G** aedeagus in ventral view **H** aedeagus in lateral view **I** aedeagus in dorsal view. Scale bars: 0.5 mm.

Head (Fig. 1E) transverse, widest across eyes; punctation coarse and sparse; interstices glossy. Eyes convex. Antennae not particularly slender, antennomere 4 approximately 3.1 times as long as broad and antennomere 10 1.7 times as long as broad. Labrum (Fig. 6A, B) with U-shaped median excision and with broad lateral projection on either side, as well as small projection on either side of median excision. Mandibles (Fig. 6A, B) each apically with bifid molar tooth, without sexual dimorphism.

Pronotum (Fig. 1E) as long as broad, moderately convex in cross section; punctation similar to that of head, but somewhat finer.

Elytra (Fig. 1E) nearly parallel-sided, wider than long; punctation coarse, well defined, and dense. Hind wings reduced. Metatarsomere I shorter than combined length of metatarsomeres II and III.

Abdomen as broad as elytra or somewhat broader than elytra; punctation dense; interstices with shallow microsculpture; posterior margin of tergite VII without palisade fringe.

**Male.** Posterior margin of tergite VIII (Fig. 6E) strongly convex; sternite VII unmodified; sternite VIII (Fig. 6F) with deep posterior excision, this excision approximately 0.4 times as long as sternite VIII; aedeagus as in Fig. 6G–I and nearly symmetric; ventral plate very weakly sclerotized; dorsal plate long and weakly curved in lateral view, extending beyond apices of parameres, dorsally with two rows of 4−5 denticles at some distance from hooked apex; parameres slender and weakly curved in lateral view; internal sac with single long sclerotized spine.

**Female.** Tergite VIII (Fig. 6C) oblong, posterior margin of strongly convex; posterior margin of sternite VIII trifurcate as in Fig. 6D.

#### Distribution and biological notes.

The species was discovered in two localities situated to southwestern Baokang, western Hubei. Some specimens were sifted from leaf litter in a mixed deciduous forest with shrubs at an altitude of 1550 m (Ye pers. comm.).

#### Etymology.

The species is named for Mao Ye, who collected some of the type specimens.

#### Comparative notes.

The geographically closest *Harpopaederus* species are *P.apfelsinicus* Willers, 2001, *P.cultellatus* Assing, 2015, and *P.multidenticulatus* Li, Solodovnikov & Zhou, 2014. *Paederusyei* is distinguished from them by the stouter pronotum, particularly by the smaller aedeagus of different morphology (dorsal plate with hooked apex; shape of the internal structure), and by the oblong female tergite VIII. For illustrations of *P.apfelsinicus* see Willers (2001: figs 16–23), of *P.cultellatus* see Assing (2015: figs 55–62), and of *P.multidenticulatus* see Li et al. (2014: fig. 2A–H).

### Paederus zhaoi

Taxon classificationAnimaliaColeopteraStaphylinidae

Yang & Peng sp. nov.

B129FEA5-E83F-5988-96A1-8C0A21DDB4EF

https://zoobank.org/B0F75D94-6D81-4D0E-8527-A3F131020410

**Figure 7.**
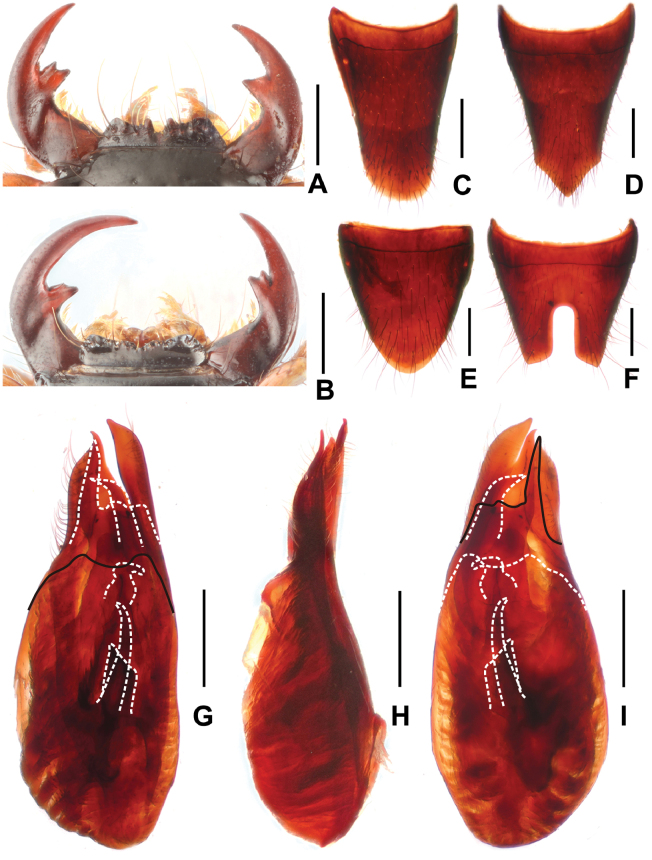
*Paederuszhaoi***A** female mouthparts **B** male mouthparts **C** female tergite VIII **D** female sternite VIII **E** male tergite VIII **F** male sternite VIII **G** aedeagus in ventral view **H** aedeagus in lateral view **I** aedeagus in dorsal view. Scale bars: 0.5 mm.

Figs 1F , 7 , 12 , 13

#### Type material.

***Holotype.*** China – **Zhejiang Prov.** • ♂; glued on a card with two labels as follows: “China: Zhejiang Prov., Zhuji City, Majian Town, Near Longmen, 29.76°N, 119.89°E, 700–1000 m, 27.IX.2023, Tie-Xiong Zhao leg.” “HOLOTYPE: *Paederuszhaoi* sp. n., Yang & Peng des. 2024” [red handwritten label]; SNUC. ***Paratypes.* Zhejiang Prov.** • 3 ♂♂, 2 ♀♀; Zhuji City, Majian Town, Near Longmen, 29.76°N, 119.89°E, 700–1000 m, 27.IX.2023, Tie-Xiong Zhao leg; SNUC.

#### Description.

Measurements (in mm) and ratios: BL 9.99–10.42, FL 4.65–4.78, HL 1.41–1.57, HW 1.56–1.82, AnL: 3.00–3.33; PL: 1.54–1.64; PW: 1.67–1.82; EL: 1.02–1.10; EW: 1.57–1.64; AW: 1.64–1.79; AL: 2.24–2.29; HL/HW: 0.85–0.92; HW/PW: 0.88–1.00; HL/PL: 0.88–0.91; PL/PW: 0.95–1.00; EL/PL: 0.66–0.68; diameter of eye: 0.33–0.37.

Habitus as in Figs 1F, 12. Coloration: head and abdomen black; antennae light brown, sometimes antennomeres 4−8 infuscate; pronotum red; elytra black with distinctly bluish hue; legs with brown femora, and with brown to light brown tibiae and tarsi.

Head (Fig. 1F) transverse, widest across eyes; punctation coarse and sparse; interstices glossy. Eyes distinctly convex. Antennae slender, antennomere 4 approximately 3.2 times as long as broad and antennomere 10 1.7 times as long as broad.

Pronotum (Fig. 1F) weakly transverse or as long as broad, strongly convex in cross section; punctures sparser and slightly finer than on head.

Elytra (Fig. 1F) trapeziform; punctation distinctly coarse, well-defined, and dense. Hind wings reduced. Metatarsomere I as long as combined length of metatarsomeres II and III.

Abdomen broader than elytra; punctation dense; interstices with distinctly transverse microsculpture; posterior margin of tergite VII without palisade fringe.

**Male.** Mandibles (Fig. 7B) each apically with small bifid molar tooth. Labrum (Fig. 7B) with shallowly concave anterior margin, and with V-shaped median excision. Posterior margin of tergite VIII (Fig. 7E) convex; sternite VII unmodified; sternite VIII (Fig. 7F) with deep posterior excision, this excision approximately 0.4 times as long as sternite VIII; aedeagus as in Fig. 7G–I, ventral plate weakly asymmetric and apically concave in ventral view; apically acute dorsal plate of distinctive shape and nearly reaching apices of parameres; parameres distinctly asymmetric, apically curved in lateral view and acute in ventral view; internal sac with three distinctive sclerotized spines and with one additional dark membranous structure.

**Female.** Left mandible (Fig. 7A) with apically bifid molar tooth; right mandible (Fig. 7A) with single-pointed middle tooth. Labrum (Fig. 7A) with U-shaped median excision and with broad lateral projection on either side, as well as small projection on either side of median excision. Tergite VIII (Fig. 7C) oblong, with convex posterior margin; posterior margin of sternite VIII (Fig. 7D) strongly convex.

#### Distribution and biological notes.

The type locality is situated to the west of Zhuji, central Zhejiang. The specimens were collected by hand on grasses and by sweep-netting of shrubs and shaking of branches both in shrub habitats with oak and bamboo at altitudes of 700–1000 m (Fig. 13).

#### Etymology.

The species is named after Tie-Xiong Zhao, who collected the type specimens.

#### Comparative notes.

The external and particularly the male sexual characters leave no doubt that this species belongs to the *P.biacutus* group. Among the species of this group, it appears to be most closely allied to *P.jianyueae* Peng & Li, 2014, with which it shares the similar morphology of the aedeagus. It is distinguished from *P.jianyueae* by the longer antennae, particularly the morphology of the aedeagus (distinctly asymmetric parameres; shape of the internal structure), and by the shape of the female tergite VIII and sternite VIII. For illustrations of *P.jianyueae* see Peng et al. (2014: figs 7, 8).

**Figures 8–13.**
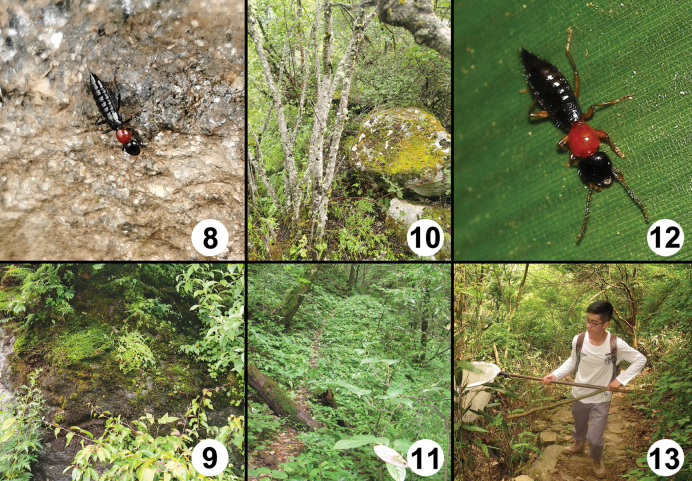
**8***Paederuschentangus* walking on a stone **9** habitat of *Paederuschentangus***10** habitat of *Paederusmirus***11** habitat of *Paederustrispinosus***12***Paederuszhaoi* walking on a blade of grass **13** Tie-Xiong Zhao collecting *Paederuszhaoi* at Majian, Zhejiang.

##### ﻿Key to the micropterous *Paederus* species of mainland China

According to recent contributions (Assing 2017; Cheng and Peng 2019; Assing 2020), three additional Chinese micropterous *Paederus* species have been described. This paper presents taxonomic and faunistic data for six new species. Therefore, the recently published key of the micropterous *Paederus* species from mainland China (Li et al. 2016) should be modified.

**Table:** 

1	Male mandibles with dorsal tooth	**2**
–	Male mandibles without dorsal tooth	**9**
2	Internal sac of aedeagus with two spines	**3**
–	Internal sac of aedeagus with one spine	**4**
3	HL/PL more than 1.00. Male right mandible with small dorsal tooth; internal sac of aedeagus with one long curved spine and one short spine. Posterior margin of female tergite VIII truncate	**P. (Gnathopaederus) szechuanus (Chapin, 1927)**
–	HL/PL no more than 0.95. Male right mandible with large dorsal tooth; internal sac of aedeagus with two short spines. Posterior margin of female tergite VIII convex	**P. (Gnathopaederus) bursavacua Willers, 2001**
4	Smaller species, length of body: 7.2 mm. Length of antenna no more than 3.0 mm	**P. (Gnathopaederus) jilongensis Li & Zhou, 2009**
–	Larger species, length of body more than 8.0 mm. Length of antenna more than 3.3 mm	**5**
5	Male sternite V–VI with shallow median impression posteriorly, this impression with weakly modified setae	**P. (Gnathopaederus) zhangmuensis Cheng & Peng, 2019**
–	Male sternite V–VI without median impression and modified setae posteriorly	**6**
6	Dorsal plate of aedeagus straight in lateral view and internal sac with one short spine	**P. (Gnathopaederus) cheni Peng & Li, 2015**
–	Dorsal plate of aedeagus weakly curved in lateral view and internal sac with one long spine	**7**
7	Male left mandible with large dorsal tooth; internal spine of aedeagus apically extending nearly to apex of dorsal plate	**P. (Gnathopaederus) furcillatus Assing, 2017**
–	Male left mandible with small dorsal tooth; internal spine of aedeagus far from reaching apex of dorsal plate	**8**
8	Smaller species, length of body: 8.3–10.5 mm. Male right mandible with small dorsal tooth; smaller aedeagus with slenderer parameres	**P. (Gnathopaederus) yunnanensis Willers, 2001**
–	Larger species, length of body 11.6–12.4 mm. Male right mandible with large dorsal tooth; larger aedeagus with stouter parameres	**P. (Gnathopaederus) xuei Peng & Li, 2015**
9	Coloration of abdomen black	**10**
–	Abdomen bicoloured (usually segments III–VI reddish and segments VII–X black)	**21**
10	Coloration of head brown. Male sternite VI with distinct median impression posteriorly	***P.lateralis* Li, Solodovnikov & Zhou, 2014**
–	Coloration of head black. Male sternite VI without median impression posteriorly	**11**
11	Male sternite VII with deep median impression posteriorly. Parameres of aedeagus very short	***P.mirus* Yang & Peng, sp. nov.**
–	Male sternite VII without impression posteriorly. Parameres long and conspicuous	**12**
12	Small species, length of body no more than 8.5 mm	***P.sinisterobliquus* Li, Zhou & Solodovnikov, 2013**
–	Large species, length of body larger than 9.0 mm	**13**
13	Maximum width of pronotum no more than 1.50 mm. Parameres of aedeagus apically distinctly curved in lateral view	***P.chentangus* Yang & Peng, sp. nov.**
–	Maximum width of pronotum more than 1.54 mm. Parameres of aedeagus apically weakly curved or straight in lateral view	**14**
14	Internal sac of aedeagus with two spines	**15**
–	Internal sac of aedeagus with three spines	**17**
15	Maximum width of abdomen more than 2.1 mm. Parameres of aedeagus slender. Female sternite IX longer	***P.parvidenticulatus* Li, Zhou & Solodovnikov, 2013**
–	Maximum width of abdomen no more than 2.0 mm. Parameres of aedeagus stout. Female sternite IX shorter	**16**
16	HL/HW more than 0.95. Aedeagus with apically hooked dorsal plate in dorsal view and two long sclerotized spines in internal sac. Female sternite VIII with elliptic depression posteriorly	***P.volutobliquus* Li, Zhou & Solodovnikov, 2013**
–	HL/HW no more than 0.90. Aedeagus with apically acute dorsal plate in dorsal view and two short sclerotized spines in internal sac. Female sternite VIII without depression	***P.nanlingensis* Peng & Li, 2016**
17	Legs with infuscate apical portion of femora. Aedeagus with apically hooked dorsal plate in dorsal view	***P.biacutus* Li, Zhou & Solodovnikov, 2013**
–	Legs with brown femora. Aedeagus with apically acute dorsal plate in dorsal view	**18**
18	Parameres of aedeagus symmetric and slender in ventral view	***P.jianyueae* Peng & Li, 2014**
–	Parameres of aedeagus asymmetric and stouter in ventral view	**19**
19	Internal sac of aedeagus with one additional dark membranous structure. Female right mandible with single-pointed middle tooth	***P.zhaoi* Yang & Peng, sp. nov.**
–	Internal sac of aedeagus without dark membranous structure. Female right mandible with small bifid molar tooth apically	**20**
20	Ventral plate of aedeagus short and broad. Posterior margin of female sternite VIII strongly convex	***P.songi* Yang & Peng, sp. nov.**
–	Ventral plate of aedeagus long and slender. Posterior margin of female sternite VIII distinctly trifurcate	***P.trispinosus* Yang & Peng, sp. nov.**
21	Aedeagus stout, with short and stout dorsal plate in dorsal view	**22**
–	Aedeagus slender, with long and slender dorsal plate in dorsal view	**24**
22	Legs reddish with infuscate apical portion of femora. Aedeagus with apically acute dorsal plate in dorsal view	***P.tibetanus* Cameron, 1928**
–	Legs yellowish brown. Aedeagus with apically convex dorsal plate in dorsal view	**23**
23	Male labrum with sinuate anterior margin. Aedeagus with short parameres. Female sternite VIII with truncate median process posteriorly	***P.describendus* Willers, 2001**
–	Male labrum with deeply excavate anterior margin. Aedeagus with long parameres. Posterior margin of female sternite VIII convex	***P.daicongchaoi* Peng & Li, 2016**
24	Dorsal plate of aedeagus not reaching apices of parameres	**25**
–	Dorsal plate of aedeagus extending beyond apices of parameres	**29**
25	Segments III–VI of abdomen reddish and with black patch in middle; elytra with pronounced impression	**P. (Harpopaederus) gottschei Kolbe, 1886**
–	Segments III-VI of abdomen pale-reddish and without black patch in middle; elytra without impression	**26**
26	Dorsal plate of aedeagus without denticles	**P. (Harpopaederus) antennocinctus Willers, 2001**
–	Dorsal plate of aedeagus with conspicuous denticles	**27**
27	Internal sac of aedeagus with one long and apically acute moderately sclerotized structure	**P. (Harpopaederus) willersi Assing, 2020**
–	Internal sac of aedeagus with one basal clip-shaped structure and one asymmetric apical structure	**28**
28	Length of aedeagus: 2.4 mm. Female tergite VIII apically narrower	**P. (Harpopaederus) deplectens Assing, 2015**
–	Length of aedeagus: 2.7 mm. Female tergite VIII apically broader	**P. (Harpopaederus) chinensis Bernhauer, 1931**
29	Dorsal plate of aedeagus without denticles	**30**
–	Dorsal plate of aedeagus with denticles	**31**
30	Length of aedeagus: 1.4 mm; internal sac without sclerotized basal structures	**P. (Harpopaederus) xui Peng & Li, 2015**
–	Length of aedeagus: 2.0 mm; internal sac with one clip-shaped, weakly sclerotized basal structure	**P. (Harpopaederus) edentulus Assing, 2015**
31	Legs with blackish metatibiae	**32**
–	Coloration of metatibiae much paler (usually yellowish)	**33**
32	Length of aedeagus: 2.4–2.7 mm, with apically stouter dorsal plate. Female sternite VIII with short median process posteriorly	**P. (Harpopaederus) apfelsinicus Willers, 2001**
–	Length of aedeagus: 2.9 mm, with apically more slender dorsal plate. Female sternite VIII with long median process posteriorly	**P. (Harpopaederus) lineodenticulatus Li & Zhou, 2007**
33	Forebody longer than 5.8 mm. Aedeagus conspicuously long (2.7 mm)	**P. (Harpopaederus) minicus Assing, 2015**
–	Forebody no more than 5.5 mm. Aedeagus shorter	**34**
34	Internal sac of aedeagus with one long, apically acute and sclerotized apical structure	**35**
–	Internal sac of aedeagus without distinctly sclerotized apical structure	**38**
35	Aedeagus 2.4 mm long, with longer apical portion of the dorsal plate	**P. (Harpopaederus) cultellatus Assing, 2015**
–	Aedeagus 1.9–2.1 mm long, with shorter apical portion of the dorsal plate	**36**
36	Dorsal plate of aedeagus with hooked apex. Posterior margin of female sternite VIII trifurcate	**P. (Harpopaederus) yei Yang & Peng, sp. nov.**
–	Dorsal plate of aedeagus with acute apex. Female sternite VIII posteriorly with median process of triangular shape	**37**
37	Tibiae usually yellowish; mandibles and shape of head without sexual dimorphisms. Aedeagus with moderately sclerotized apical internal structure	**P. (Harpopaederus) agnatus Eppelsheim, 1889**
–	Tibiae distinctly infuscate basally; mandibles and shape of head with pronounced sexual dimorphisms. Aedeagus with strongly sclerotized apical internal structure	**P. (Harpopaederus) konfuzius Willers, 2001**
38	Femora brown. Dorsal plate of aedeagus with 20 small denticles; parameres apically weakly curved in lateral view	**P. (Harpopaederus) multidenticulatus Li, Zhou & Solodovnikov, 2014**
–	Femora bicoloured. Dorsal plate of aedeagus with several large denticles; parameres apically hooked in lateral view	**39**
39	Head transverse (HL/HW: 0.90). Dorsal plate of aedeagus with short apical portion. Female sternite VIII with strongly convex posterior margin	**P. (Harpopaederus) brevior Li, Zhou & Solodovnikov, 2014**
–	Head weakly transverse (HL/HW: 0.99). Dorsal plate of aedeagus with long apical portion. Female sternite VIII with long median process posteriorly	**P. (Harpopaederus) gracilacutus Li & Zhou, 2007**

## Supplementary Material

XML Treatment for Paederus chentangus

XML Treatment for Paederus mirus

XML Treatment for Paederus songi

XML Treatment for Paederus trispinosus

XML Treatment for Paederus (Harpopaederus) yei

XML Treatment for Paederus zhaoi

## ﻿Additional information

### Author ORCIDs

Yi Yang https://orcid.org/0000-0003-1132-9103

Sophie Chen https://orcid.org/0009-0007-3396-9810

Zhong Peng https://orcid.org/0000-0001-5959-1536

### Data availability

All of the data that support the findings of this study are available in the main text.
